# Accumulation of free nuclei denotes defective phagocytic capacity of macrophages and occurs after infection with *Listeria monocytogenes* and lymphocytic choriomeningitis virus

**DOI:** 10.3389/fimmu.2025.1621608

**Published:** 2026-01-27

**Authors:** Theresa Charlotte Christ, Justa Friebus-Kardash, Michael Bergerhausen, Abdelrahman Elwy, Hossam Abdelrahman, Lisa Holnsteiner, Tobias Tertel, Elisa Wiebeck, Ilka Geuer, Alexander Gerbaulet, Philipp Alexander Lang, Karl Sebastian Lang

**Affiliations:** 1 Institute of Immunology, Medical Faculty, University of Duisburg-Essen, Essen, Germany; 2 Department of Nephrology, University Hospital Essen, University Duisburg-Essen, Essen, Germany; 3 Abalos Therapeutics GmbH, Düsseldorf, Germany; 4 Institute for Transfusion Medicine, University Hospital Essen, University Duisburg-Essen, Essen, Germany; 5 Institute for Immunology, Faculty of Medicine, Technische Universität (TU) Dresden, Dresden, Germany; 6 Institute of Molecular Medicine II, Heinrich-Heine-University, Düsseldorf, Germany

**Keywords:** phagocytosis, macrophages, Listeria monocytogenes, LCMV, interferon-gamma (IFN-γ), clodronate

## Abstract

Efficient phagocytosis of pathogens is a key effector function of the innate immune system. Impaired phagocytic activity can result in uncontrolled pathogen proliferation and life-threatening infections. However, reliable methods to detect early dysfunction of the phagocytic system *in vivo* are limited. Here, we used a mouse model of *Listeria monocytogenes* infection to determine blood parameters which correlate with limited macrophage function. We found that lack of macrophages led to accumulation of nuclei in the blood. Further analysis of nuclei revealed that these nuclei were released from bone marrow-derived cells. Macrophage-depleted mice and interferon-gamma-deficient mice, which are known to have reduced phagocytotic capacity, showed increased amounts of free nuclei. This was associated with lethal outcome and occurrence of acute hepatopathy in these mice after *Listeria monocytogenes* infection. Our findings highlight a simple and noninvasive method to assess macrophage phagocytic function *in vivo*, which should be assessed in further murine and human studies as a tool for predicting host vulnerability to infection.

## 1 Introduction

Macrophages are professional phagocytic cells that play a central role in maintaining tissue homeostasis and orchestrating immune responses. Through phagocytosis, macrophages eliminate infected or dying cells, invading pathogens, cancer cells, and cellular debris. This process is not only essential for controlling infection and preventing the spread of disease but also for promoting tissue repair and regeneration following injury or inflammation (1, 2). Timely detection of localized pathogens by macrophages is mediated by different phagocytic transmembrane surface receptors which recognize particles > 0.5 µm and ingest them into phagosomes (3, 4). Macrophages are equipped with scavenger receptors (SRs), which allow them to specifically bind and phagocytose pathogens. SRs are a structurally heterogeneous superfamily of proteins (5). There are several classes of SRs, which are classified based on their nucleotide sequence alignment and protein structure (6, 7). One particular type of such scavenger receptors are phosphatidyl serine receptors that allow macrophages to recognize phosphatidyl serine, which in apoptotic cells is released from the inner to the outer plasma (8, 9). Binding of pathogens or cell debris to SRs can induce Src and Syk phosphorylation followed by triggering phagocytosis of the pathogen.

Thus, macrophages counteract pathogens and cell debris. After internalization, microbes proceed to phagosomes that further fuse with multivesicular bodies or lysosomes (2, 10). Within these highly acidic compartments, pathogens are killed by diverse anti-microbial mechanisms such as digestion by proteases and antimicrobial peptides or degradation by reactive oxygen and nitrogen species, lysozymes and lactoferrin (2, 10). These mechanisms allows liver resident macrophages (Kupffer cells) and splenic macrophages (red pulp and marginal zone macrophages), which are both associated with the endothelium and reach into the blood vessel lumen, to efficiently capture and eliminate blood invading pathogens within the first minutes to hours after infection and as a consequence to limit the systemic spread of the pathogen (1, 2, 11–14) Previous murine studies as well as our work provided evidence that the phagocytic capacity of neutrophils and macrophages and the TNF-α signaling pathway are the key players in prevention of invasion and dissemination of pathogens in the blood that may play a protective role for the development of sepsis after bacterial and viral infections (10, 11, 15–19).

Several inherited deficiencies are described that affect the number of phagocytic cells, the phagocytic capacity or the digestion of pathogens after phagocytosis (20). These diseases typically result in increased incidence, dissemination and severity of infections (20). Similarly, limited phagocytosis was discovered in the AIDS stage of HIV infection, which could explain the high susceptibility to opportunistic infections (21). However, other interventions and diseases that influence phagocytic capacity are not well studied and we consider this knowledge gap to be due to limited possibilities to analyze the phagocytic capacity *in vivo*.

In this study, we aimed to measure cellular debris and/or cell fragments which accumulate in the blood of mice with reduced phagocytic capacity of macrophages. We identified a specific population of free nuclei that arise in murine blood due to disturbed phagocytosis of macrophages and their accumulation was associated with lethal outcome after infection with *Listeria monocytogenes* and Lymphocytic Choriomeningitis Virus (LCMV).

## 2 Methods

### 2.1 Mice

H2B-GFP mice were described recently (22). For induction, mice were fed doxycycline-containing food pellets (2 g/kg; Ssniff Spezialdiäten) for 1 week. Mice with the same genotype and gender as experimental animals, but fed with normal food, were used as a negative control. *Ifng^-/-^* mice were purchased from Jackson Laboratories. Vav1-iCre x Gt(Rosa)26Sor (eYFP) mice were bred as a cross between the breeding line B6.Cg-Commd10Tg(Vav1-icre)A2Kio/J and B6.129X1-Gt(ROSA)26Sortm1(EYFP)Cos/J. Both strains were provided by Jackson Laboratories (#008610, #006148). All mice used were maintained on a C57BL/6 background. All experiments were performed in single ventilated cages. Animal experiments were authorized by the Veterinäramt Nordrhein-Westfalen (Düsseldorf-Essen, Germany) and in accordance with the German law for animal protection or according to institutional guidelines at the Ontario Cancer Institute of the University Health Network. Mice were sex- and age-matched and used in experiments at 6–20 weeks of age.

### 2.2 ALT, AST and LDH measurement

The analysis of liver enzymes alanine aminotransferase (ALT) and aspartate aminotransferase (AST), as well as lactate dehydrogenase (LDH), from mouse serum was performed at the central laboratory of the University Hospital Essen.

### 2.3 Listeria monocytogenes infection and titers

*Listeria monocytogenes* (ATCC strain 43251) bacteria were the kind gift of Klaus Pfeffer (Institute for Medical Microbiology and Hospital Hygiene, Heinrich-Heine-University of Düsseldorf, Universitätsstr. 1, D-40225 Düsseldorf, Germany) and were maintained on heart infusion agar as previously described (23). Unless otherwise indicated, mice were infected intravenously with 1x10^2^ CFU of *L. monocytogenes*.

For several experiments, the bacteria were inactivated with UV light and inactivation was confirmed using a dilution smear on agar plates. The CFSE labeling of 1x10^6^ CFU *L. monocytogenes* was performed with the CFSE Cell Division Tracker Kit from BioLegend (Cat: 423801) following the manufacturer’s instructions. Bacterial counts were determined from homogenized organs, plated in serial dilutions on brain–heart infusion agar.

### 2.4 LCMV infection and plaque assay

The LCMV strain WE was generously provided by Rolf Zinkernagel from the Institute of Experimental Immunology (ETH Zurich, Switzerland) and propagated in BHK cells (24). Mice were infected intravenously with 2x10^5^ PFU of LCMV WE. Viral titers were quantified using a plaque-forming assay on MC57 fibroblasts as previously described (25).

### 2.5 Cell depletion

For granulocyte and monocyte depletion, 100 μg per mouse of anti-Gr-1 antibody (anti-mouse Ly6G/Ly6C, clone RB6-8C5, Bio X Cell, Lebanon, USA) was administered 48 hours prior to the measurement of free nuclei by flow cytometry. CD71^+^ cell depletion was achieved by administering 200 μg per mouse of anti-CD71 antibody (InVivoMAb anti-mouse CD71 [TfR1], clone R17 217.1.3/TIB-219, Bio X Cell, Lebanon, USA) on days -4, -3, and -2 prior to nuclei analysis. All depletion experiments were performed via intravenous (i.v.) injection.

### 2.6 Clodronate treatment

Clodronate Liposomes and Control Liposomes were purchased from Liposoma. They typically contain a clodronate concentration of approximately 5 mg/ml. If not otherwise mentioned, macrophages were depleted by injecting 250 µl of Clodronate Liposomes per mouse intraperitoneally (i.p.) 1 day before nuclei measurement.

### 2.7 Bone marrow irradiation

One day before or one day after irradiation, mice were treated with clodronate. For bone marrow depletion, mice were irradiated with 9.5 Gy. On the following day, bone marrow from a donor was isolated under sterile conditions and injected i.v.

### 2.8 Isolation of nuclei

The isolation was performed with the Minute™ Single Nucleus Isolation Kit for Tissues/Cells from Invent following the manufacturer’s instructions.

### 2.9 Flow cytometry

For detection of nuclei, blood was incubated with DAPI for 5 minutes. Anti-Lamin B1 antibody (clone EPR8985(B), Abcam) and anti-CD47 antibody (clone miap301, eBioscience) were used for staining. Erythrocytes were lysed for 7 min before washing. Cells were resuspended in 200 μl FACS buffer containing 1:100 AccuCheck Counting Beads (Cat# PCB100, Thermo Fisher). Absolute numbers were counted according to the manufacturer instructions. Nuclei appeared as DAPI high events. Free CFSE-labeled *Listeria monocytogenes* and the H2B signal were detected in FL-1.

### 2.10 Multiplex assay

Serum cytokines were analyzed with the LEGENDplex™ (BioLegend, San Diego, CA, USA) according to the instructions and recommendations of the manufacturer. Serum cytokines were analyzed with the LEGENDplex™ Mouse Anti-Virus Response Panel (13-plex) (BioLegend, San Diego, CA, USA); LEGENDplex is a multiplex immunoassay based on fluorescence-encoded beads and flow cytometry measurements. Briefly, before the assay, all samples were thawed at room temperature and centrifuged for 5 min at 1000 x g. The samples were then pre-diluted 1:2 and incubated for 2 h with monoclonal capture antibody coated beads. Following this, the beads were washed and incubated for 1h with biotin-labelled detection antibodies. Finally, the samples were incubated with streptavidin-PE for 30 min. After staining, beads were acquired by FACS Fortessa flow cytometry (BD Bioscience, Franklin Lakes, NJ, USA) using the BD FACSDiva™ software (BD Bioscience, Franklin Lakes, NJ, USA). To determine cytokine concentrations for each sample, we used the LEGENDplex Data Analysis Software (BioLegend, San Diego, CA, USA) and followed the manufacturer’s protocol to extrapolate the results from standard curves.

### 2.11 Histology

Histological analyses were performed on snap-frozen or formalin-fixed tissues as previously described (10). For staining different cells types standard antibodies were used (BioLegend, BD Biosciences). For staining of LCMV, the clone VL4 was used.

### 2.12 Image flow cytometry

Images of the nuclei were acquired using the AMNIS ImageStreamX Mark II Flow Cytometer (Luminex, Seattle, WA, USA) after the target population had been sorted using the BD FACSDiscover™ S8. Data analysis was performed using the IDEAS software.

### 2.13 Preparation of bone marrow-derived macrophages

Bone marrow was harvested from the femurs and tibias of C57BL/6 mice and red blood cells were lysed using the RBC lysis buffer (PAN Biotech, Cat# P10-90100). The remaining cells were collected by centrifugation and resuspended at a density of 1.5×10^6^ cells/well in complete RPMI medium with 10% FCS supplemented with or without 25 ng/mL M-CSF on a 24-well plate. Cultures were incubated at 37°C with 5% CO_2_.

### 2.14 Latrunculin A treatment of peritoneal macrophages

Peritoneal macrophages were obtained by peritoneal lavage. Mice were euthanized according to institutional guidelines, and the peritoneal cavity was flushed with sterile PBS containing 2% FBS. The lavage fluid was collected, centrifuged, and resuspended in complete medium (DMEM supplemented with 10% FBS and penicillin/streptomycin). Cells were seeded in culture plates and maintained for 24 h at 37°C and 5% CO_2_ to allow adherence before experiments were initiated. Peritoneal macrophages were treated with latrunculin A at the indicated concentrations for 30 min at 37°C (26) prior to incubation with DAPI-stained nuclei. Latrunculin A was purchased from Invitrogen.

### 2.15 Statistical analysis

Data are presented as means with ± SEM. Differences between two groups were determined using the unpaired Student’s t test. Survival was assessed by Kaplan-Meier analysis, and p values were determined by the log-rank test. All p values were two-tailed and p values ≤ 0.05 were considered statistically significant. All statistical analyses were performed with GraphPad Prism version 6 (GraphPad Software, Inc., La Jolla, CA, USA).

## 3 Results

### 3.1 Macrophages are critical for rapid clearance of bacteria, *Listeria monocytogenes*, from the bloodstream

**Figure 1:**
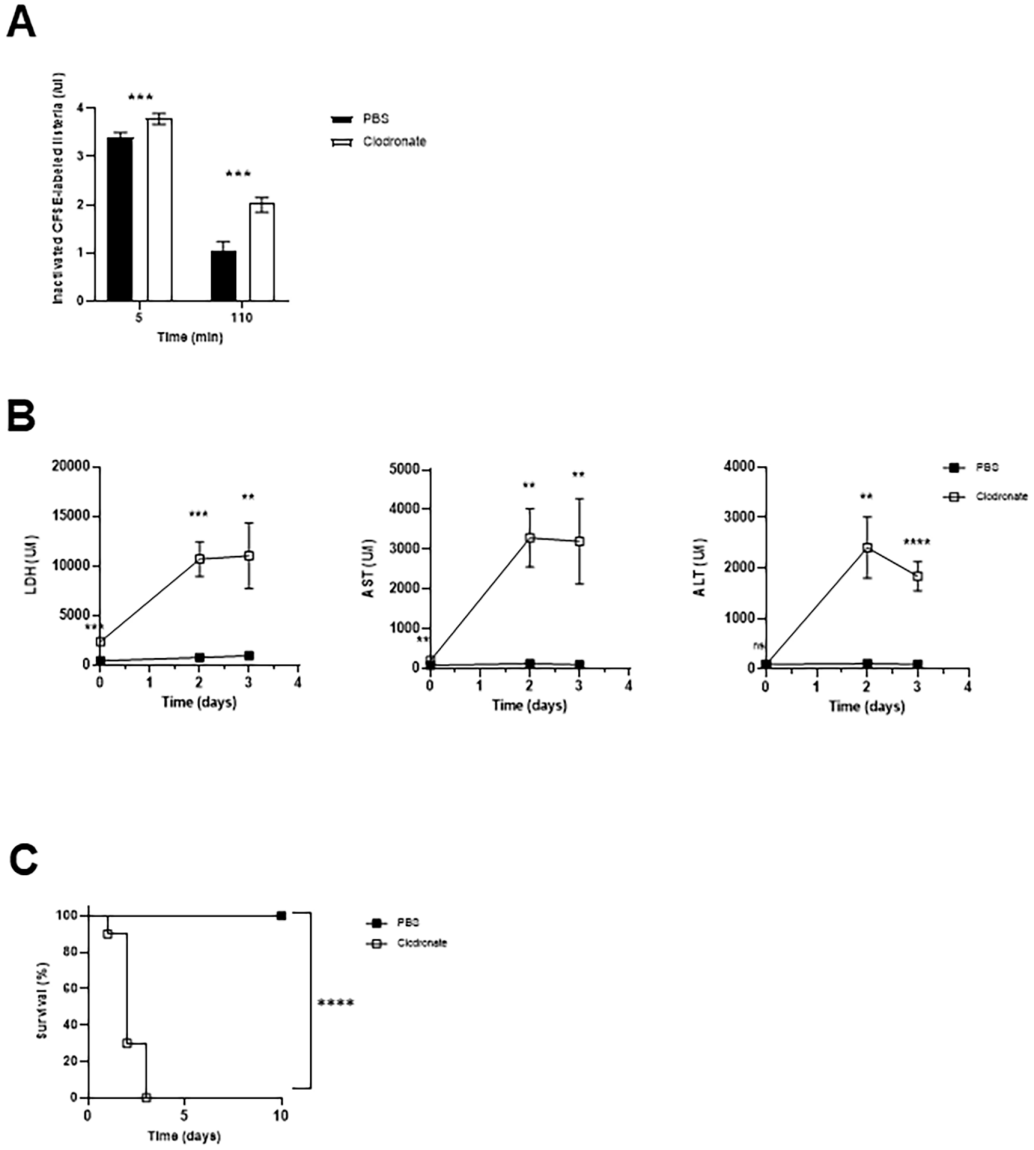
Depletion of macrophages using clodronate treatment prevents mice from controlling bacterial infection with *Listeria monocytogenes*. **(A)** Comparison of the uptake of inactivated CFSE (carboxyfluorescein succinimidyl ester)-labeled *Listeria monocytogenes* between clodronate treated C57BL/6 mice (n=6) and C57BL/6 mice treated with PBS as a control (n=6). Uptake of inactivated *Listeria monocytogenes* by phagocytic immune cells was determined as CFSE-positive cells per µl blood. Clodronate treatment was performed by intraperitoneal injection of 250 µl of clodronate liposomes 24 hours before injection of inactivated CFSE-labeled *Listeria monocytogenes*. 1x10^6^ CFU of inactivated CFSE-labeled *Listeria monocytogenes* were intravenously administered per mouse. Uptake of inactivated CFSE-labeled *Listeria monocytogenes* was measured using flow cytometry of the whole blood collected at 3 minutes and 2 hours after injection of inactivated *Listeria monocytogenes*. **(B)** Serum levels of liver enzymes were quantified at the indicated time points after infection with *Listeria monocytogenes* of clodronate and PBS treated animals. C57BL/6 mice were pretreated with 250 µl of clodronate liposomes (n=6) or PBS (n=6) and then infected intravenously with 1x10^2^ CFU of *Listeria monocytogenes* 24 hours after pretreatment. **(C)** Survival analysis during intravenous bacterial infection with 1x10^2^ CFU of *Listeria monocytogenes* comparing clodronate pretreated C57BL/6 mice (n=10) and C57BL/6 mice pretreated with PBS as control (n=10). Data are shown as mean ± SEM and are pooled from 2 independent experiments. **p=0.01; ***p=0.001; ****p=0.0001; **(A, B)** unpaired two-tailed Student’s t test; **(C)** Log-rank (Mantel-Cox).

Efficient phagocytosis of pathogens by macrophages, monocytes, and granulocytes is required to eliminate bacterial pathogens and to control acute bacterial infections (1, 2). To directly assess the role of macrophages in early bacterial clearance, we depleted macrophages using clodronate liposomes and subsequently injected C57BL/6 mice with 1 × 10^6^ CFSE-labeled, inactivated *Listeria monocytogenes* (**Figure 1A**). As early as 3 minutes post-injection, we observed a significant reduction in bacterial uptake in clodronate-treated mice compared to controls. Over the course of 120 minutes, control mice efficiently cleared the inactivated bacteria from circulation, whereas macrophage-depleted mice exhibited a markedly impaired clearance. Specifically, the levels of CFSE-labeled *Listeria monocytogenes* remaining in the blood were approximately tenfold higher in clodronate-treated mice, demonstrating the essential role of macrophages in the rapid removal of blood-borne bacterial pathogens (**Figure 1A**). Next, we aimed to determine the physiological relevance of the limited clearance due to diminishment of phagocytes by clodronate. For this purpose, C57BL/6 mice pretreated with clodronate liposomes or PBS were intravenous infected with 10^2^ CFU of *Listeria monocytogenes*. Serum levels of liver enzymes such as transaminases and lactate dehydrogenase (LDH) significantly increased already after two days post infection with *Listeria monocytogenes* in clodronate treated mice (**Figure 1B**). In control mice, liver enzyme levels remained stable over three days post infection (**Figure 1B**). According to liver failure seen in biochemical tests of clodronate treated C57BL/6 mice they quickly died within the first three days after infection with *Listeria monocytogenes*, while all control mice survived (**Figure 1C**). These observations emphasize that deficiency of macrophages induced by clodronate pretreatment resulted in persistence of *Listeria monocytogenes* in C57BL/6 mice with subsequent organ failure after acute infection.

### 3.2 Free nuclei accumulated in the blood of macrophage depleted mice

**Figure 2:**
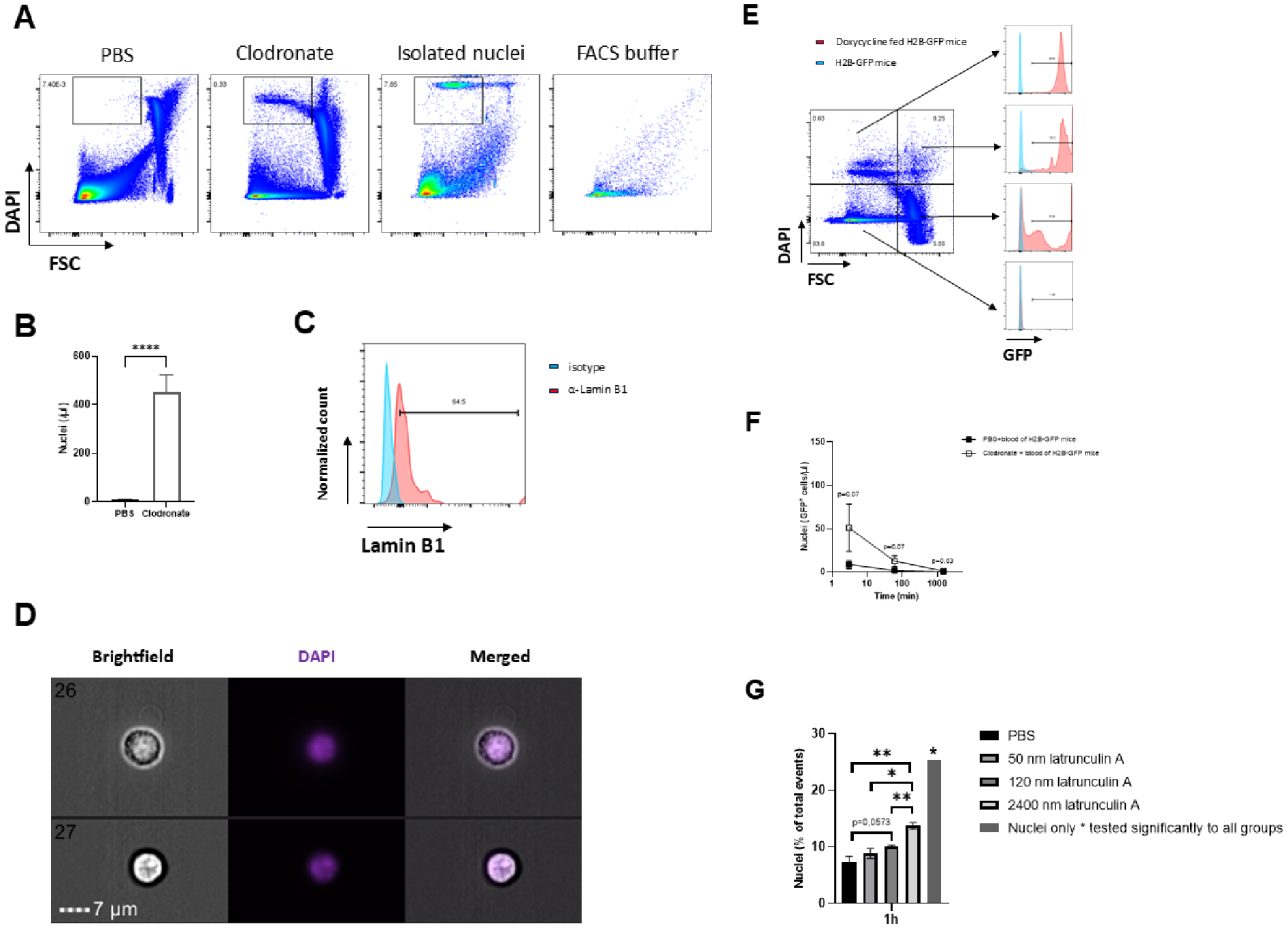
Free particles detected in the blood circulation are considered to be free nuclei whose accumulation is caused by disrupted removal by macrophages. **(A)** Identification of free nuclear particles using flow cytometry based on their small size in forward scattering and their positivity when stained with DAPI (4’,6-diamidino-2-phenylindole), a classic marker that binds to DNA (deoxyribonucleic acid) in the cell nucleus. Nuclei were isolated from bone marrow derived cells of naive C57BL/6 mice using Minute™ Single Nucleus Isolation Kit for Tissues/Cells and stained for DAPI and analyzed by flow cytometry. We saw a similar pattern in flow cytometry corresponding to free nuclei at 24 hours after treatment of C57BL/6 mice with 250 µl of clodronate liposomes that are commonly used to deplete phagocytic cells, in particular macrophages (n=6). Indeed, we did not observe any free nuclei particles in flow cytometry staining when C57BL/6 mice were treated with 250 µl of PBS as a control solution. Buffer-only controls were included to identify non-cellular events and buffer-derived particles (n=6). **(B)** A significant accumulation of free nuclei in whole blood of C57BL/6 mice was observed only after pretreatment with clodronate at day -1 (n=6) and not with PBS, indicating that phagocytosis is predominantly involved in the clearance of free nuclei from the blood circulation (n=6). **(C)** In order to further characterize free nuclei particles, whole blood of C57BL/6 mice pretreated at day -1 with 250 µl of clodronate liposomes was stained for the protein lamin B1, as a part of nuclear lamina matrix. Isotype antibody was used as control. **(D)** Representative images of the free particles deriving from the sorted FCS low, DAPI positive population. For the analysis C57BL/6 mice were intraperitoneally treated with clodronate. Blood was collected at 24 hours after treatment, stained with DAPI and the target FCS low, DAPI positive population was subsequently sorted using the BD FACSDiscover™ S8. Images were acquired with the AMNIS ImageStreamX Mark II Flow Cytometer (Luminex, Seattle, WA, USA) and are shown as brightfield, DAPI and merged channels. Data analysis was performed using the IDEAS software. Scale bar = 7 µm. **(E, F)** Gt(ROSA)26Sor^tm1(rtTA*M2)Jae^/Col1a1^tm7(tetO-HIST1H2BJ/GFP)Jae^, shortly called H2B-GFP mice exhibited GFP (green fluorescent protein) positive histones H2B in all cells of the body after previous induction with doxycycline. Food pellets containing doxycycline (2 g/kg of doxycycline hyclate; ssniff Spezialdiäten GmbH, Soest, Germany) were given to H2B-GFP mice at day -7 in order to induce the expression of GFP-labeled H2B histones. H2B-GFP mice which were on a normal diet were defined as wild type mice. 500 µl blood obtained from H2B-GFP mice was intravenously injected into C57BL/6 mice that were pretreated with 250 µl of clodronate liposomes (n=6) or PBS (n=6) 24 hours before the injection. Free nuclei particles were quantified in flow cytometry analysis at 3 minutes, 1 hour and 24 hours after blood transfer from H2B-GFP mice. Free circulating nuclei sized, DAPI positive particles were positive for GFP in flow cytometry analysis, suggesting that GFP positive H2B histones were integrated in the detected free nuclei particles. Depletion of phagocytic immune cells due to clodronate pretreatment was associated with a trend toward lower clearance of free nuclei expressing GFP positive H2B histones derived from the blood of H2B-GFP mice among clodronate pretreated C57BL/6 mice compared to PBS treated controls. **(G)** Accumulation of free nuclei after inhibition of actin polymerization by Latrunculin A in peritoneal macrophages. Peritoneal macrophages were treated *in vitro* with different concentrations of latrunculin A or PBS for 30 min and subsequently incubated with DAPI-stained nuclei for 1 h (n=3). The percentage of free nuclei among total events was determined by flow cytometry. The nuclei-only condition served as a control (n=1). Data derived from two independent experiments with consistent results are given as mean ± SEM. ****p=0.0001; **(B)** unpaired two-tailed Student’s t test; **(F)** unpaired one-tailed Student’s t test. "*" indicates p < 0.05 and "**" indicates p < 0.01.

Given the crucial role of macrophages in eliminating pathogens, we next investigated whether macrophage dysfunction also impairs the clearance of endogenous cellular debris from the bloodstream. Mechanisms of cell death such as apoptosis occurring in all body cells induces formation of cell fragments in particular free nuclei that can be quickly eliminated by macrophages under healthy physiological conditions (1, 2). Thus, lack of macrophage function would result in accumulation of free nuclei and cell debris in the blood circulation. The size and shape of the nucleus vary across the different cell types and change during developmental progression, cellular differentiation, and aging (27). In mice, neuronal nuclei have an average diameter of 10.6 µm (28), whereas the nuclei of cortical and medullary lymphocyte are of smaller size, measuring about 4.9 µm (29). They are easily stainable with the deoxyribonucleic acid (DNA) dye DAPI (4’,6-diamidino-2-phenylindole) (30). We could easily measure nuclei by flow cytometry when samples of highly concentrated nuclei isolated from bone marrow derived cells of naive C57BL/6 mice were stained with DAPI (**Figure 2A**). After staining the whole blood obtained from clodronate pretreated mice with DAPI, we observed events that were highly positive for DAPI and of very small size in the forward scatter highly likely corresponding to free nuclei (**Figure 2A**).

Further quantification of free nuclei by flow cytometry revealed a significant increase of free nuclei in C57BL/6 mice after depletion of macrophages via clodronate liposomes in comparison to C57BL/6 mice receiving PBS as a control substance that again underlines the relevance of macrophages for the removal of free nuclei from blood circulation (**Figure 2B**). To provide further evidence that the population identified in the absence of macrophages belongs to nuclei, we performed staining for the nuclear membrane marker protein, Lamin B1 (**Figure 2C**). Flow cytometry staining of the DAPI positive population for Lamin B1 revealed a moderate expression (**Figure 2C**).

To assess the morphology and texture of the specific FSC low, DAPI-positive population, we analyzed representative events using the ImageStream system after cell sorting (**Figure 2D**). The objects measured approximately 6–8 µm in diameter and displayed round shapes (**Figure 2D**). In all cases, compact, strongly fluorescent DAPI signals were entirely enclosed within the brightfield-visible structures (**Figure 2D**). The combination of size, shape, internal granularity and distinct DNA staining suggested that the sorted population most likely represents cell nuclei.

Next, we depleted macrophages via clodronate liposome application in doxycycline treated and untreated H2B-GFP mice (**Figure 2F**). Previous treatment with doxycycline induced green fluorescent protein (GFP) expression on histone H2B in all nuclei of body cells (**Figure 2F**). Hence, DAPI positive population identified in doxycycline treated H2B-GFP mice that underwent subsequent clodronate treatment expressed high levels of GFP (**Figure 2E**). To address the question of whether the GFP positive nuclei which accumulate in macrophage depleted H2B-GFP mice could be removed by macrophages, we transferred blood from the clodronate pretreated H2B-GFP mice into C57BL/6 mice, which were either treated with clodronate or left untreated (**Figure 2F**). We observed a trend toward higher amounts of free nuclei with GFP-labeled H2B histones among clodronate pretreated C57BL/6 mice in comparison to the PBS pretreated C57BL/6 mice at 3 minutes (8.56 vs. 51.0, p=0.07) and 60 minutes (1.61 vs. 12.37, p=0.066) after transfer of the blood from H2B-GFP mice, but the difference between the two groups did not reach statistical significance (**Figure 2F**). This finding suggests a possible relationship between the absence of phagocytic cells, in particular macrophages, and the slower elimination of GFP positive nuclei.

To test whether the accumulation of free nuclei is related not only to a quantitative reduction in macrophage numbers but also to qualitative differences in their phagocytic function, peritoneal macrophages were treated *in vitro* with latrunculin A, an inhibitor of actin polymerization and subsequently phagocytosis (26). After 1 h incubation with DAPI-stained nuclei, the percentage of free nuclei increased in a concentration-dependent manner (**Figure 2G**). Low concentrations showed a trend toward higher levels, whereas intermediate and high concentrations induced a clear and significant increase compared to PBS controls (**Figure 2G**). The nuclei-only condition displayed the highest proportion of nuclei and served as a reference (**Figure 2G**).

In a separate set of experiments, we depleted granulocytes and monocytes using anti-Gr-1 antibody in C57BL/6 mice that did not lead to accumulation of free nuclei in our flow cytometry assay (**Supplementary Figure 1**).

Additionally, we observed mild expression of CD47 signal on free nuclei particles (**Supplementary Figure 2A**). Moreover, the percentage of Apotracker Green positive events within the FSC low, DAPI positive population was low with approximately 10% indicating that it is unlikely that the free particles belong to the classical apoptotic bodies that are characterized by high expression of phosphatidylserine that is bound by the Apotracker Green (**Supplementary Figure 2B**).

In conclusion, we found that macrophage deficiency, but not dysfunction of granulocytes and monocytes, resulted in accumulation of free nuclei in murine blood.

### 3.3 Number of free nuclei in blood circulation is an independent surrogate marker for macrophage function

**Figure 3:**
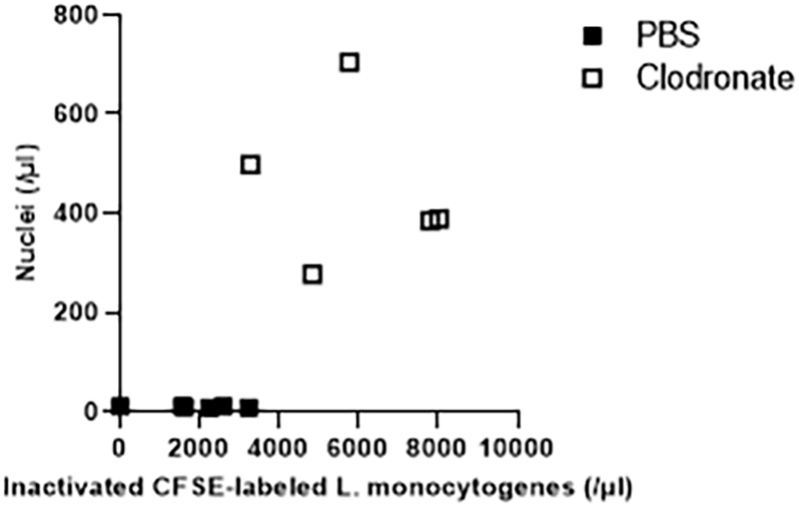
Measurement of free nuclei in the blood circulation as a surrogate parameter for macrophage dysfunction. We used an alternative approach to demonstrate that the accumulation of free nuclei depends on the impairment of phagocytic capacity. Inactivated CFSE (carboxyfluorescein succinimidyl ester)-labeled *Listeria monocytogenes* are mostly eliminated by phagocytic cells, in particular macrophages. For this purpose, 10^6^ CFU of inactivated CFSE-labeled *Listeria monocytogenes* were intravenously administered per mouse and flow cytometry measurement was performed after 3 minutes (n=5). Depletion of phagocytic cells using 250 µl of clodronate liposomes injected intraperitoneally one day before resulted not solely in the development of free nuclei, but in reduced clearance of inactivated CFSE-labeled *Listeria monocytogenes* with subsequent accumulation of inactivated *L. monocytogenes* in whole blood. An increase of free nuclei correlated with elevation of inactivated CFSE-labeled *L. monocytogenes* in C57BL/6 mice which were intraperitoneally pretreated with 250 µl of clodronate liposomes 24 hours before flow cytometry analysis. Data are combined from 2 to 3 independent experiments with consistent results.

We detected an accumulation of free nuclei in the absence of macrophages using our flow cytometry assay. Then, we wondered how this parameter correlates with other known factors that determine the outcome of infection. To gain insights, we depleted macrophages in mice with clodronate, injected them with inactivated CFSE-labeled *Listeria monocytogenes*, and afterwards determined free nuclei and inactivated *Listeria monocytogenes*. Free nuclei were not detectable in control mice, whereas we could still identify some CFSE-labeled *Listeria monocytogenes* (**Figure 3**). On the other hand, macrophage depleted mice showed high levels of free nuclei and high amounts of CFSE-labeled *Listeria monocytogenes* (**Figure 3**). Macrophage depletion increased the amount of free nuclei 47-times. In contrast, the difference of free inactivated CFSE-labeled *Listeria monocytogenes* was only 3.2-times higher for the clodronate treatment than for the control group receiving PBS (**Figure 3**). Therefore, we concluded that measurement of free nuclei by flow cytometry is more robust and sensitive for assessing macrophage function than the alternative approach of evaluating clearance of inactivated CFSE-labeled *Listeria monocytogenes* by macrophages.

### 3.4 Free nuclei derived from hematopoietic cells, predominantly erythropoietic stem cells

**Figure 4:**
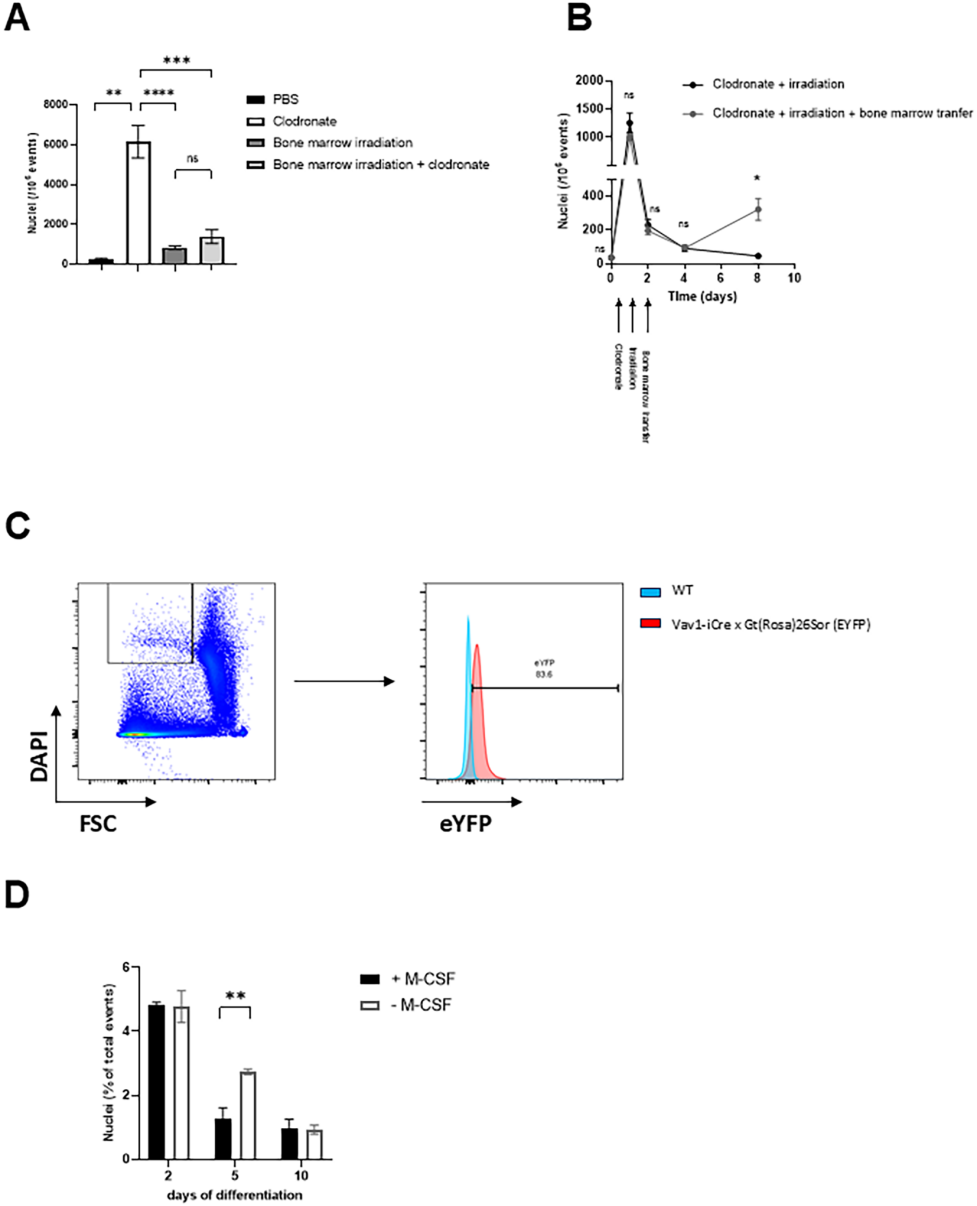
Free nuclei derive from bone marrow derived cells, predominantly erythropoietic stem cell with a high turnover. **(A)** Depletion of phagocytic cells using 250 µl of clodronate liposomes was performed in naive C57BL/6 mice and C57BL/6 mice that underwent irradiation 48 hours before intraperitoneal treatment with clodronate. Irradiation was performed with 9.5 Gray. Accumulation of free nuclei particles was only visible in non-irradiated C57BL/6 mice pretreated with clodronate that displayed free circulating nuclei sized particles, that were DAPI positive in flow cytometry measurement conducted at 24 hours thereafter (n=6). Indeed, free nuclei particles fully disappeared in bone marrow irradiated C57BL/6 mice independently of the following treatment with clodronate (n=6). **(B)** First, all C57BL/6 mice were treated with 250 µl of clodronate liposomes at day 0 to deplete phagocytes and underwent irradiation at day 1. Next, bone marrow derived cells isolated from naive C57BL/6 mice were transferred at day 2 via intravenous injection into those C57BL/6 mice that were pretreated with clodronate and irradiated (n=3). The control group received PBS instead of bone marrow derived cells (n=3). Transfer of bone marrow derived cells was related to the appearance of free nuclei particles in the blood of C57BL/6 mice compared to the control mice without bone marrow transfer. **(C)** All hematopoietic cells of Vav1-iCre x Gt(Rosa)26Sor (eYFP) mice express the fluorescent dye, eYFP (Enhanced Yellow Fluorescent Protein). Vav1-iCre x Gt(Rosa)26Sor (eYFP) mice and wild type mice were pretreated with 250 µl of clodronate liposomes at day -1 before analyzing their blood with our flow cytometry assay. The representative histogram shows positivity for eYFP of the population of free small sized, DAPI positive nuclei particles obtained from macrophage depleted Vav1-iCre x Gt(Rosa)26Sor (eYFP) mice. **(D)** Quantification of the free nuclei in bone marrow cultures treated with or without M-CSF. Supernatants were collected at day 2, 5, and 10, stained with DAPI, and analyzed by flow cytometry to quantify the amount of free nuclei. Data are presented as the percentage of free nuclei in relation to the total number of events. Data derived from two independent experiments with consistent results are given as mean ± SEM. *p=0.05; **(B)** unpaired two-tailed Student’s t test. ns: not significant (p > 0.05), **: p < 0.01, ***: p < 0.001, ****: p < 0.0001.

Next, we aimed to clarify the origin of free nuclei. We considered bone marrow derived hematopoietic cells as potential candidates because of high turnover and short lifespan of these cell types. To gain deeper insights, C57BL/6 mice were first treated with clodronate liposomes to disturb phagocytosis and thereafter one group of clodronate pretreated C57BL/6 mice underwent bone marrow irradiation and the other group did not, serving as a control (**Figure 4A**). Free nuclei particles fully disappeared from the blood circulation after bone marrow irradiation of mice with a depleted phagocytic system, while animals that did not experience bone marrow irradiation after clodronate treatment displayed, as expected, high amounts of freely circulating nuclei (**Figure 4A**).

In the next experiment, transfer of bone marrow derived hematopoietic cells isolated from naive C57BL/6 mice were transferred into C57BL/6 mice that were previously pretreated with clodronate and irradiated (**Figure 4B**). This procedure promoted the occurrence of free nuclei (**Figure 4B**). Indeed, those C57BL/6 mice from the control group that were only pretreated with clodronate and irradiated without receiving subsequent bone marrow transfer developed no detectable free nuclei (**Figure 4B**).

To support our observations in an independent model, we depleted macrophages using clodronate in Vav1-iCre x Gt(Rosa)26Sor (eYFP) mice that exhibited expression of the fluorescent dye, eYFP (Enhanced Yellow Fluorescent Protein) in all their hematopoietic cells. The distinct population of free nuclei obtained from the whole blood of Vav1-iCre x Gt(Rosa)26Sor (eYFP) mice after macrophage depletion using our flow cytometry assay was positive for eYFP, giving an additional hint that free nuclei detected in our assay derived from hematopoietic cells (**Figure 4C**).

To investigate the cellular mechanisms that could explain the occurrence of free nuclei detected *in vivo*, we established *in vitro* bone marrow–derived cultures and analyzed how macrophage differentiation influenced the number of free nuclei over time. We performed *in vitro* assays using bone marrow-derived macrophages (BMDMs) treated with or without GM-CSF to quantify nuclear counts under different conditions (**Figure 4D**). The total number of free nuclei declined progressively during the culture period (**Figure 4D**). At day 2, both groups exhibited high numbers of free nuclei (**Figure 4D**). At day 5, free nuclei levels decreased in both groups, but the decrease was more pronounced in the GM-CSF–treated cultures (**Figure 4D**). At day 10, we measured the lowest levels of free nuclei in both groups (**Figure 4D**).

From these data, we concluded that the natural turnover of bone marrow derived cells leads to release of free nuclei, which are usually quickly phagocytosed by macrophages.

### 3.5 Deficiency of IFN-γ was associated with an increase of free nuclei and led to an acute hepatopathy with a fatal outcome after bacterial infection with *Listeria monocytogenes*

**Figure 5:**
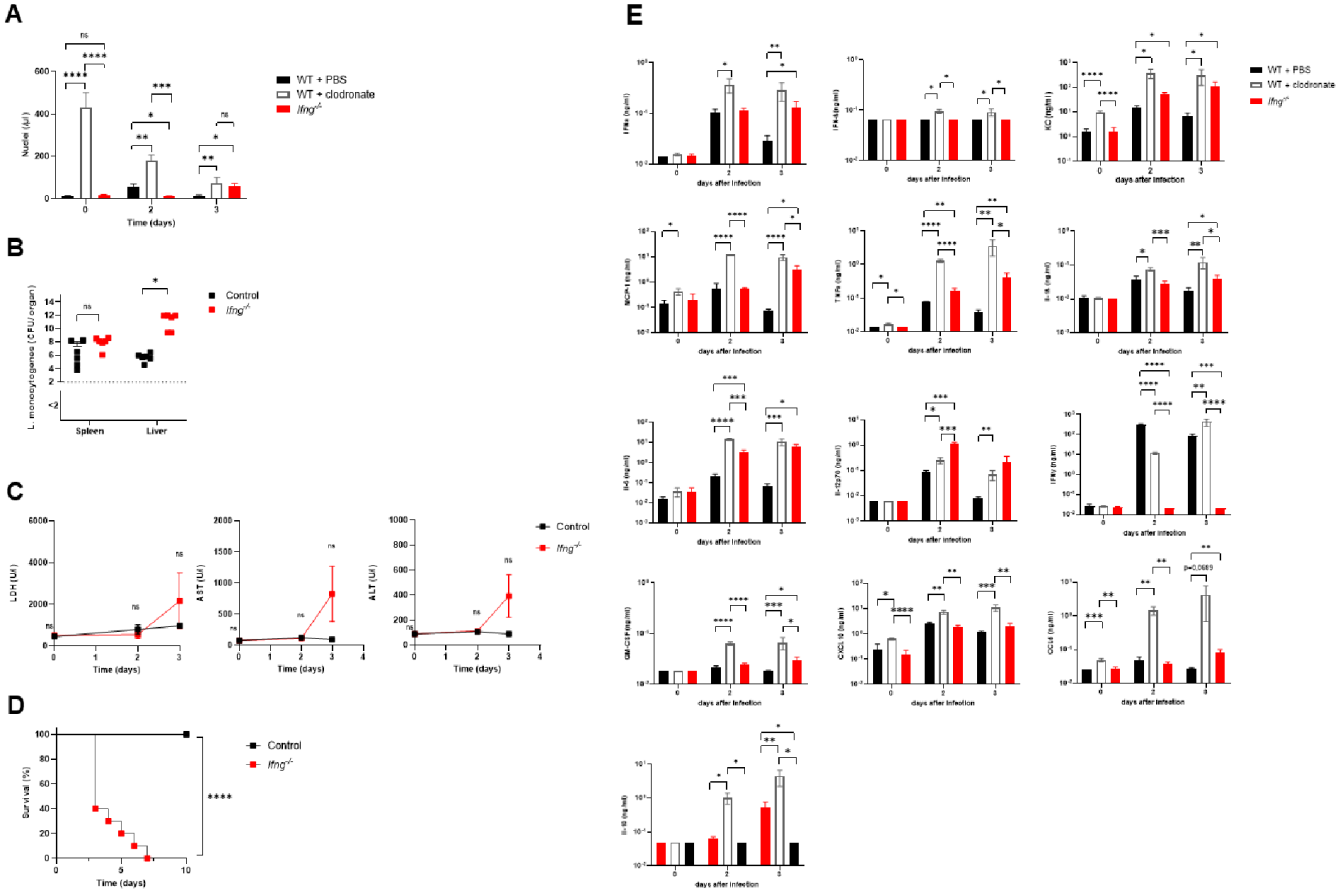
IFN-γ deficiency results in impaired pathogen phagocytosis and leads to a fatal course of bacterial infection with *Listeria monocytogenes*. **(A)** Detection of free nuclei particles among C57BL/6 mice pretreated with clodronate or PBS and IFN-γ deficient mice that were intravenously infected with 1x10^2^ CFU of *Listeria monocytogenes* (n=6-8). The amount of free nuclei was quantified by flow cytometry in the whole blood of the animals on days 0, 2, and 3 post bacterial infection. C57BL/6 mice received 250 µl of clodronate liposomes or PBS as a control solution i.p. 24 hours before bacterial injection. **(B)** Concentrations of *Listeria monocytogenes* in liver and spleen tissue of IFN-γ deficient and WT mice were assessed in colony forming units on day 4 after intravenous infection with 1x10^2^ CFU of *Listeria monocytogenes* (n=6). **(C)** Serum concentrations of liver enzymes were measured in IFN-γ deficient and WT mice at specified time points following intravenous infection with 1x10^2^ CFU of *Listeria monocytogenes* (n=6). **(D)** Reduced survival rates after intravenous infection with 1x10^2^ CFU of *Listeria monocytogenes* in IFN-γ deficient mice (n=10) compared to WT mice (n=10). **(E)** Serum levels of IFN-α, IFN-β, CXCL1 (KC), CCL2 (MCP-1), TNF-α, IL-1β, IL-6, IL-12p70, IFN-γ, GM-CSF, CXCL10 (IP-10), CCL5 (RANTES), and IL-10 were measured in the serum by multiplex cytokine assay in wild-type mice treated with clodronate liposomes and IFN-γ deficient mice on days 0, 2, and 3 after infection (n=4-10). Data is shown as mean ± SEM and is pooled from 2–3 independent experiments. *p=0.05; **p=0.01; ***p=0.001; ****p=0.0001; **(A, B)** unpaired two-tailed Student’s t test; **(C)** unpaired one-tailed Student’s t test; **(D)** Log-rank (Mantel-Cox); **(E)** unpaired two-tailed Student’s t test. “ns” indicates “not significant” (p > 0.05).

IFN-γ, primarily produced by T lymphocytes, NK cells and macrophages during acute infection, is a key regulator of innate immune function. One of its central roles is to enhance the phagocytic capacity of macrophages by promoting their activation, increasing expression of phagocytic receptors, and boosting antimicrobial mechanisms essential for efficient clearance of pathogens (31, 32). Therefore, we attempted to measure the accumulation of free nuclei in IFN-γ deficient mice. Naive *Ifng*^-/-^ mice showed similar low amounts of free nuclei when compared with wild type mice (**Figure 5A**). This is in line with virtually absent levels of IFN-γ in the absence of infection. After acute intravenous bacterial infection with 10^2^ CFU of *Listeria monocytogenes*, we detected an accumulation of free nuclei in IFN-γ deficient mice whereas the amount of free nuclei remained hardly unchanged in wild type mice (**Figure 5A**). Bacterial titers revealed accelerated spread of *Listeria monocytogenes* in *Ifng^-/-^* mice (**Figure 5B**). In line *Ifng^-/-^* mice showed a worsening of liver function compared to wild type mice and *Ifng^-/-^* mice died quickly after infection (**Figures 5C, D**). These data reinforce the idea that impaired phagocytosis is directly linked to accumulation of free nuclei.

Next, to compare the functional consequences of impaired phagocytosis due to IFN-γ deficiency with those resulting from complete macrophage depletion, we analyzed systemic cytokine and chemokine profiles following intravenous *L. monocytogenes* infection in IFN-γ-deficient and wild-type mice that had been pretreated with clodronate liposomes or PBS (**Figure 5E**). Serum concentrations of a variety of pro- and anti-inflammatory cytokines were measured at baseline (day 0) and at day 2 and 3 post infection (**Figure 5E**). *Ifng-/-* mice and clodronate-treated wild-type and exhibited a pronounced systemic cytokine response with a marked increase in a number of proinflammatory mediators (**Figure 5E**). Concentrations of TNF-α, IL-1β and IL-6 significantly increased at day 2 and remained elevated at day 3 in comparison to the wild-type mice receiving PBS pre-treatment (**Figure 5E**). The highest concentration of different proinflammatory cytokines was observed among clodronate-treated mice. Similarly, levels of IL-12p70 and GM-CSF were significantly higher among *Ifng-/-* mice and clodronate treated mice than in wild type mice pretreated with PBS serving as controls (**Figure 5E**).

Chemokine production followed a similar trend with a robust induction of MCP-1 (CCL2), CXCL10 (IP-10), KC (CXCL1) and CCL5 (RANTES) in both *Ifng-/-* clodronate-treated with the highest levels achieved at day 2 or 3. Furthermore, the highest production of MCP-1 and KC, that are responsible for the recruitment of monocytes and neutrophils, was seen among clodronate pretreated wild type mice (**Figure 5E**). In contrast to the *Ifng-/-* mice with the lack of IFN-γ release, clodronate pretreated mice had high levels of IFN-γ (**Figure 5E**).

Interestingly, this hyperinflammatory reaction was accompanied by a parallel rise of the anti-inflammatory cytokine IL-10 at day 2 and 3 among *Ifng-/-* mice and wild type mice pretreated with clodronate when comparing to the controls (**Figure 5E**). The simultaneous upregulation of proinflammatory cytokines (including TNF-α, IL-1β, IL-6, IL-12p70, GM-CSF) and IL-10 indicated a dysregulated systemic immune response, having features of hyperinflammation and compensatory anti-inflammatory signaling (**Figure 5E**).

In conclusion, our data indicated that the accumulation of free nuclei (**Figure 5A**) correlates with uncontrolled bacterial spread (**Figure 5B**) and a lethal outcome in *Ifng-/-* mice following *Listeria monocytogenes* infection (**Figure 5D**). In addition, a pronounced systemic cytokine response characterized by markedly elevated pro- and anti-inflammatory mediators and chemokines was detected after systemic infection with Listeria monocytogenes among *Ifng-/-* mice and *Ifng-/-* mice (**Figure 5E**).

### 3.6 LCMV infection of IFN-γ deficient mice was associated with an increase of free nuclei and limited viral clearance in the liver

**Figure 6:**
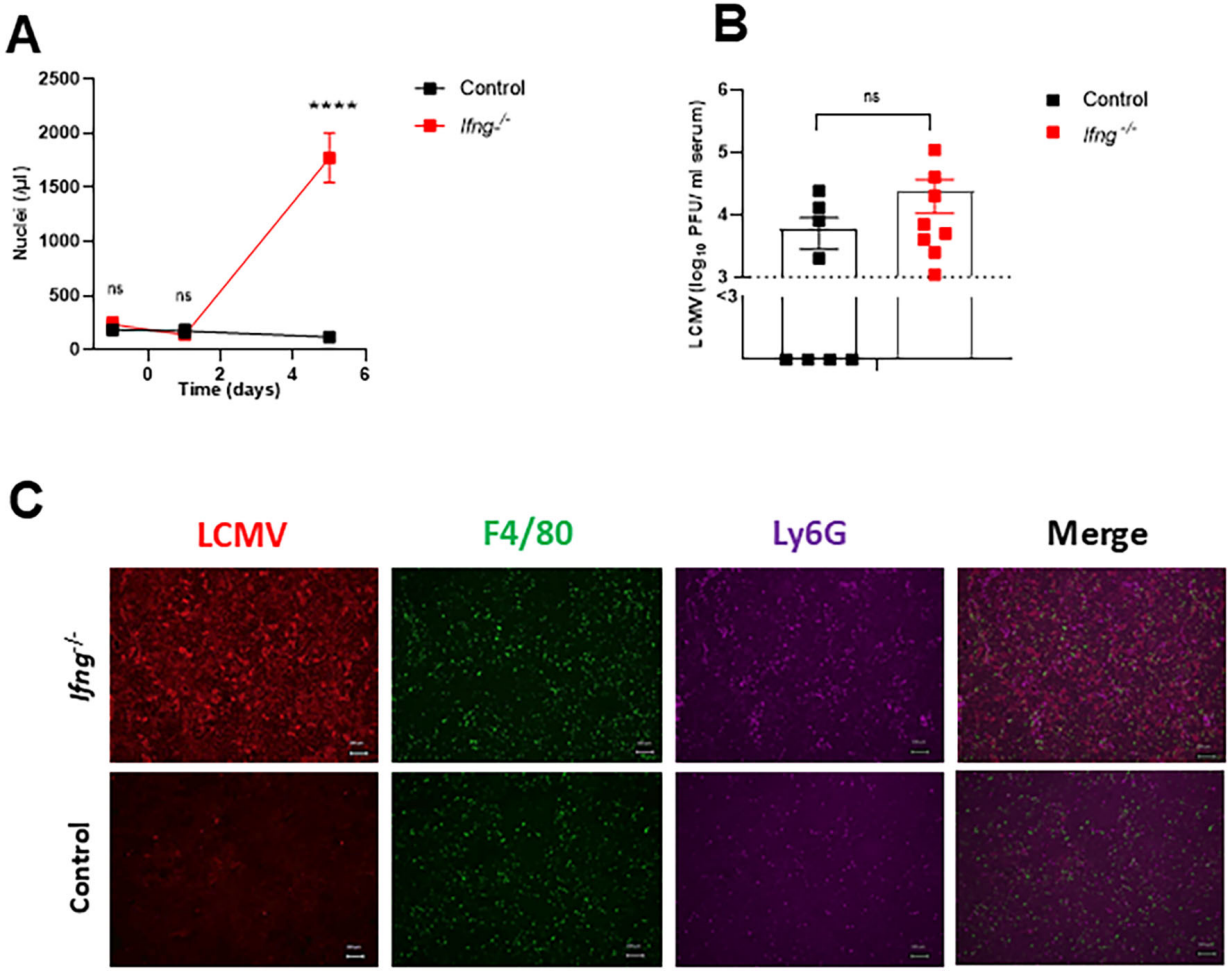
Deficiency of IFN-γ inhibits clearance of LCMV in the liver after acute viral infection. **(A)** Release of free nuclei particles in IFN-γ deficient and WT mice after acute LCMV infection. Intravenous LCMV infection with LCMV WE (high dose, 2x10^5^ PFU per mouse) was conducted in IFN-γ deficient and WT mice. Nuclei were analyzed by flow cytometry in whole blood samples from IFN-γ deficient and WT mice on days -1, 1, and 5 after infection (n=6). **(B)** Viral titers were determined by plaque assay in serum of IFN-γ deficient and WT mice on day 5 after infection with 2x10^5^ PFU of LCMV WE (n=8). **(C)** Immunofluorescence analysis stained for LCMV nucleoprotein, F4/80 and Ly6G positive cells was performed on snap-frozen liver sections from IFN-γ deficient and WT mice on day 5 after LCMV infection. Scale bar = 100 μm; one representative out of 6 is shown. Fluorescent microscopy images were captured at 10x magnification using Keyence BZ-9000E microscope. Data is shown as mean ± SEM and is pooled from 2–3 independent experiments. ****p=0.0001; **(A)** unpaired two-tailed Student’s t test; **(B)** unpaired one-tailed Student’s t test. “ns” indicates “not significant” (p > 0.05).

Next, we addressed the role of IFN-γ deficiency in acute viral infection using lymphocytic choriomeningitis virus (LCMV). LCMV is known to induce a strong T cell response, and therefore high levels of IFN-γ are measurable. Besides its antiviral activity, IFN-γ increases macrophage phagocytosis, which is essential to limit viral dissemination and clear debris from virus infected cells. Similarly to bacterial infection with *Listeria monocytogenes*, there was a rise of free nuclei in LCMV infected IFN-γ deficient mice (**Figure 6A**). At the time, the particles were highly detectable there was no significant increase of virus detected in the serum (**Figure 6B**). However, increased amounts of free nuclei correlated with enhanced viral antigen expression in the liver (**Figure 6C**). The data demonstrate that deficiency of IFN-γ prevents mice from controlling acute viral infection, most likely because of the lack of enhancement of phagocytic capacity of macrophages during infection.

## 4 Discussion

Macrophages play a pivotal role in the elimination of pathogens through phagocytosis. To assess the phagocytic capacity of macrophages we established a new simple assay quantifying DAPI positive, small sized particles by flow cytometry corresponding to nuclei particles freely circulating in the blood. We identified an accumulation of these free particles after depletion of macrophages with clodronate in C57BL/6 mice. Analysis of isolated nuclei derived from C57BL/6 mice by our flow cytometry assay revealed a population that strongly resembled the free particles, implying that detected particles correspond to free nuclei. Furthermore, free particles were stained positively for H2B histones suggesting that histones belong to the components of the free particles. Blood transfer from H2B-GFP mice expressing GFP positive H2B histones after doxycycline induction into clodronate pretreated C57BL/6 mice resulted in a trend toward higher amounts of GFP positive free particles, emphasizing their nuclear origin. Free nuclei particles disappeared in our assay after bone marrow irradiation of clodronate pretreated mice, whereas the occurrence of free nuclei particles was seen again after bone marrow transfer into irradiated clodronate pretreated C57BL/6 mice, indicating that free circulating nuclei particles mainly derived from hematopoietic cells with high turnover. We measured an increase of free nuclei particles in our assay after viral infection with LCMV and bacterial infection with *Listeria monocytogenes* of IFN-γ knockout mice. These mice were not able to eliminate LCMV and *Listeria monocytogenes* compared to wild type mice that might be related to limited macrophage function and impaired phagocytic capacity due to IFN-γ deficiency. During infection with *Listeria monocytogenes*, this uncontrolled proliferation of bacteria was accompanied by a dysregulated cytokine response, suggesting maladaptive systemic immune activation as a result of increased bacterial load.

Currently, there are several approaches to measure phagocytosic capacity of macrophages *in vitro* (33–36). Most assays assess the internalization of specific target particles by macrophages (33–36). In general, two different types of phagocytic targets are utilized in *in vitro* assays for macrophage phagocytosis, model cells like apoptotic mammalian cells, antibody-opsonized sheep red blood cells, bacteria or yeast and proteins of interest coated to beads (33–39). As an example of phagocytosis assays studying phagocytosis on cell types, the use of fluorescent strains of Escherichia coli is common to investigate the phagocytosis of bacterial cells (35). On the other hand, the use of synthetic target ligands attached to polystyrene, latex or glass particles via non-specific adsorption enables researchers to consider the involvement of a specific ligand-receptor interaction in the macrophage mediated phagocytosis (33, 36, 38, 39). Recently, Joffe et al. developed a macrophage phagocytosis *in vitro* assay with reconstituted membrane-coated target particles to which a broad variety of synthetic proteins of interest can bind that provides an opportunity to identify isolated molecular interactions leading to receptor-mediated phagocytosis by macrophages (33). However, the principle of these previously widely used *in vitro* protocols differs from our *in vivo* method using nuclei particles easily measured by fluorescence cytometry as a surrogate marker of impairment of phagocytosis process in mice. Thus, our approach pursues another objective than the above-mentioned *in vitro* assays focusing on detection of phagocytosis defects. Xu and colleagues described an assay analyzing the efficiency of macrophage phagocytosis of cancer cells *in vitro* and *in vivo* (40). For the *in vivo* part a mouse subcutaneous xenograft tumor model was constructed and xenograft tumors consisting of CFSE-labeled lung cancer cell line were digested (40). To analyze the phagocytosic capacity of macrophages *in vivo*, macrophages deriving from subcutaneous tumors were stained with rat anti-mouse CD11b and F4/80 antibodies, sorted by flow cytometry and phagocytosis efficiency was determined as the percentages GFP positive F4/80^+^ CD11b^+^ cells in total F4/80^+^ CD11b^+^ macrophages (40).

The exact origin of the free particles detected by our flow cytometry remains a subject of debate while our results support the nuclear origin of the free particles released in the case of impaired phagocytosis of macrophages. However, we first speculated that the free particles identified in our flow cytometry assay might represent neutrophil extracellular traps (NETs). NETs are extracellular, web-like decondensed nuclear or mitochondrial DNA structures containing histones and cytosolic and granule proteins (41, 42). The interplay between the CXC receptor 2 expressed on neutrophils and the corresponding CXC ligand 2 located on endothelial cells occurs during infection and contributes to chemotaxis resulting in release of high amounts of NETs from neutrophils proteins (41, 42). Neutrophil elastase and myeloperoxidase contribute to the breakdown of chromatin and nuclear envelope and granular mixing in the NET vacuole, afterwards mature NETs are extruded because of the rupture of the outer membrane of neutrophils (41, 42). The main function of NETs is trapping and neutralization of pathogen proteins (41, 42). Formation of NETs was also associated with cell death and cell damage (43). Our experiments on H2B-GFP mice with GFP-labeled H2B histones that exhibited DAPI positive particles in our flow cytometry assay showed that H2B histone belongs to the components of the free particles. Likewise, NETs carry H2B histones and DNA as its major components (42). Indeed, we did not find an accumulation of free particles in our flow cytometry assay performed at 48 hours after the intravenous application of the anti-Gr-1 antibody to the C57BL/6 mice for the purpose of the depletion of neutrophil granulocytes and monocytes underlining that neutrophils and monocytes as well as NETs do not seem to be involved in the production of free particles detected by our assay. Hence, the appearance of free particles seems to be the only characteristic for the depletion of macrophages and impairment of macrophage phagocytosis. On the other hand, macrophages are able to produce extracellular traps, so called METs, in order to attract and immobilize pathogens (44). In the case of METs we would expect net-like formations in image flow cytometry analysis. But image flow cytometry analysis of free particles revealed a round form of the particles most likely compatible with regular cell nuclei that excludes the possibility that METs might participate in the formation of free particles identified in our flow cytometry assay. Additionally, our results provided several hints that the turn-over of hematopoietic cells, predominantly erythropoietic stem cells, instead of direct destruction of macrophages leads to the occurrence of free particles that argues against the theory that free particles might be parts of METs deriving from damages macrophages. Alternatively, we hypothesized that free particles might belong to exosomes. Exosomes are small endosome-derived membrane vesicles of 40–150 nm that are secreted by diverse cell types and are composed of proteins, lipids and nucleic acids with histones such as RNA and DNA (45). Exosomes arise from multivesicular bodies that fuse with cell membrane and release exosomes into the extracellular space by exocytosis (45). Exosomes help intercellular communication by transferring their functional components to recipient cells (45). Production of exosomes is elevated upon infection, in particular sepsis (45). So far, double-stranded DNA was evident only in tumor-derived exosomes (46). On the other hand, it is conceivable that DAPI positive free particles might act as cell-free DNA that serves as damage-associated molecular pattern. However, we saw that the free particles were of much larger size in flow cytometry that did exosomes or cell-free DNA according to the size of a free nucleus that again suggests nuclear origin of the corresponding free particles measured by our assay. However, we cannot fully exclude that the free particles containing DNA detected by our assay might derive from other kind of extracellular vesicles such as microvesicles that display larger size than exosomes.

Macrophages are responsible for the clearance of apoptotic cell material (47). One of the hallmarks of apoptotic cell death is the exposure of phosphatidylserine on the outer leaflet of cytoplasmic membrane in a caspase-dependent manner that can be recognized by corresponding phagocytic receptors for phosphatidylserine on macrophages (47). Expression of the transmembrane protein CD47 on cell surface and its interplay with SIRP**-α** prevents cells from phagocytic degradation by macrophages (48). Vice versa CD47 expression was shown to be down regulated on apoptotic cells (33). However, free particles detected by our assay did not lose CD47 expression. The expression of the apoptosis marker annexin V was also low in the DAPI positive population of free particles. Thereby, we concluded that the free particles did not derive from apoptotic debris.

In our work, we clearly showed that deficiency of IFN-γ limited the clearance of bacterial and viral pathogens resulting in accumulation of free nuclei in our assay. Occurrence of high amounts of free nuclei upon infection with *Listeria monocytogenes* and LCMV in IFN-γ deficient mice might be mostly related to dysfunction of macrophages. IFN-γ is the principal cytokine activating macrophages (31). Interaction of PAMPs with corresponding receptors on macrophages induces secretion of interleukin-12 by macrophages that subsequently force CD4 positive Th1 effector cells to secrete IFN-γ (31). In the context of positive feedback, IFN-γ attaches to the IFN-γ-receptor located on macrophages amplifying phagocytotic capacity (31, 49). In line with our observations, previous reports demonstrated that IFN-γ deficient mice had substantial defects in defense against bacterial and viral pathogens including *Listeria monocytogenes* and LCMV while in naive state these mice having aberrant IFN-γ expression showed no overt development defects of immune system (49). The number of macrophages was described not to be affected in IFN-γ knock out mice but the mice exhibited disrupted production of antimicrobial products by macrophages and reduced expression of MHC class II antigens on macrophages explaining their increased susceptibility to bacterial infections (49, 50). Several viruses such as LCMV require not only interferon type I response but also parallel activation of interferon type II pathways for successful clearance of virus particles at an early stage of infection (49). Therefore, nonfunctional IFN-γ genes might prevent effective antiviral activity upon murine LCMV infection (49).

Under infectious conditions, IFN-γ was shown to inhibit emergency granulopoiesis through activation of SOCS3 in granulocyte-macrophage progenitors and consequently restricting the production of the STAT3 as a critical transcription factor for the G-CSF-mediated emergency granulopoiesis (51). Thus, in IFN-γ deficient mice that were infected with Mycobacterium bovis, Mycobacterium tuberculosis or Toxoplasma gondii, an increase of infection-induced emergency granulopoiesis with a rise of neutrophils in bone marrow and peripheral blood was demonstrated (51). Based on this knowledge from previous studies on IFN-γ deficient mice, we might conceive that the occurrence of free particles observed in IFN-γ deficient mice upon infection with *Listeria monocytogenes* in our assay might be attributed to the increased development of neutrophil granulocytes and the subsequent increased turnover of neutrophil granulocytes in the bone marrow of IFN-γ deficient mice. In steady state, in the absence of infection, the IFN-γ deficiency did not strongly affect granulopoiesis that might lead to comparable low numbers of free particles in IFN-γ deficient mice and control mice. However, we followed an alternative hypothesis explaining the lack of free particles under steady state conditions and the appearance of free particles upon infection with *Listeria monocytogenes* in IFN-γ deficient mice. The enhanced release of IFN-γ is triggered by infection and is responsible for further augmentation of phagocytic capacity of macrophages during infection process (31). In this way, failure to phagocyte due to the lack of IFN-γ manifested during acute infection reflecting the elevation of free particles in IFN-γ deficient mice at day three post infection with *Listeria monocytogenes*. In naïve state the deficiency of IFN-γ has less relevance for phagocytosis, which is why phagocytic capability of IFN-γ deficient mice was comparable with those of C57BL/6 mice according to the low production of free nuclei particles in both mice lines.

In clodronate pretreated mice the highest number of free particles was detected at steady state, before infection. Interestingly, at the follow-up of infection with *Listeria monocytogenes* the number of free particles decreased in clodronate pretreated mice. This observation contradicts the presumption that the emergency granulopoiesis might be the source for the accumulation of free particles upon infection because in this case we would expect the further increase of the amounts of free particles upon infection *Listeria monocytogenes* in clodronate pretreated mice due to the increased turnover of neutrophils. We think that the decrease of free particles in the course of infection in clodronate pretreated mice might be mostly related to the weakening of the clodronate effect on macrophages over time and restitution of the phagocytic capacity of macrophages.

Our *in vitro* results show that the accumulation of free nuclei is related to impaired phagocytosis by macrophages. Phagocytosis is an actin-dependent process (52). Inhibition of actin dynamics by latrunculin A demonstrated that besides of the reduced macrophage numbers observed in the clodronate experiments, the functional impairment of phagocytic capacity of macrophages might contribute to the increased proportion of free nuclei. Based on our results, the number of macrophages as well as the functionality were crucial for the clearance of nuclei. In addition, the faulty activation of macrophages due to an IFN-γ deficiency led to reduced clearance of cell nuclei, which was subsequently associated with acute hepatitis and a fatal outcome after bacterial infection with *Listeria monocytogenes*. Our findings were consistent with observations from several human studies indicating that insufficient or delayed phagocytosis and clearance of pathogens by macrophages leads to exogenous pathogen invasion and systemic infection and might result in development and progression of sepsis (53–57). We believe that our findings warrant further evaluation of our *in vivo* flow cytometry assay measuring free nuclei in human studies on sepsis patients as a biomarker for disturbed phagocytic rate and potential predictor for sepsis exacerbation.

To conclude, we showed that the absence of the phagocytic function of macrophages resulted in overwhelming growth of pathogens and correlated with accumulation of free nuclei in the blood of mice.

## Data Availability

The raw data supporting the conclusions of this article will be made available by the authors, without undue reservation.

## Supplementary material

Supplementary Figure 1 The appearance of free nuclei particles was attributed only to depletion of macrophages and not of neutrophils and monocytes. C57BL/6 mice were treated intraperitoneally with 250 µl of clodronate liposomes in order to deplete macrophages and monocytes 24 hours before measurement of free nuclei particles by flow cytometry (n=3). 100 μg per mouse of anti-Gr-1 antibody (anti-mouse Ly6G/Ly6C antibody, clone RB6-8C5, Bio X Cell, Lebanon, USA) were applied i.v. for depletion of neutrophil granulocytes and monocytes 48 hours before measurement of free nuclei by flow cytometry (n=3). Results are presented as mean ± SEM.

Supplementary Figure 2 Free nuclei were unlikely to represent apoptotic debris, as indicated by mild CD47 expression and low Apotracker Green positivity. **(A)** The whole blood of C57BL/6 mice pretreated at day -1 with 250 µl of clodronate liposomes was stained using an antibody against surface protein CD47, which is involved in inhibition of phagocytosis. The representative histogram shows positivity for CD47 of the population of free small sized, DAPI positive nuclei particles. Isotype antibody was used as control. **(B)** The whole blood of C57BL/6 mice pretreated at day -1 with 250 µl of clodronate liposomes was stained using Apotracker Green. The representative histogram shows positivity for Apotracker Green of the population of free small sized, DAPI positive nuclei particles. Unstained cells were used as negative control.
