# Supplementary figures and images for: Accumulation of free nuclei denotes defective phagocytic capacity of macrophages and occurs after infection with Listeria monocytogenes and lymphocytic choriomeningitis virus
Source: Front Immunol. 2026 Jan 27;16:1621608. doi: 10.3389/fimmu.2025.1621608 (PMC12888213; doi:10.3389/fimmu.2025.1621608)

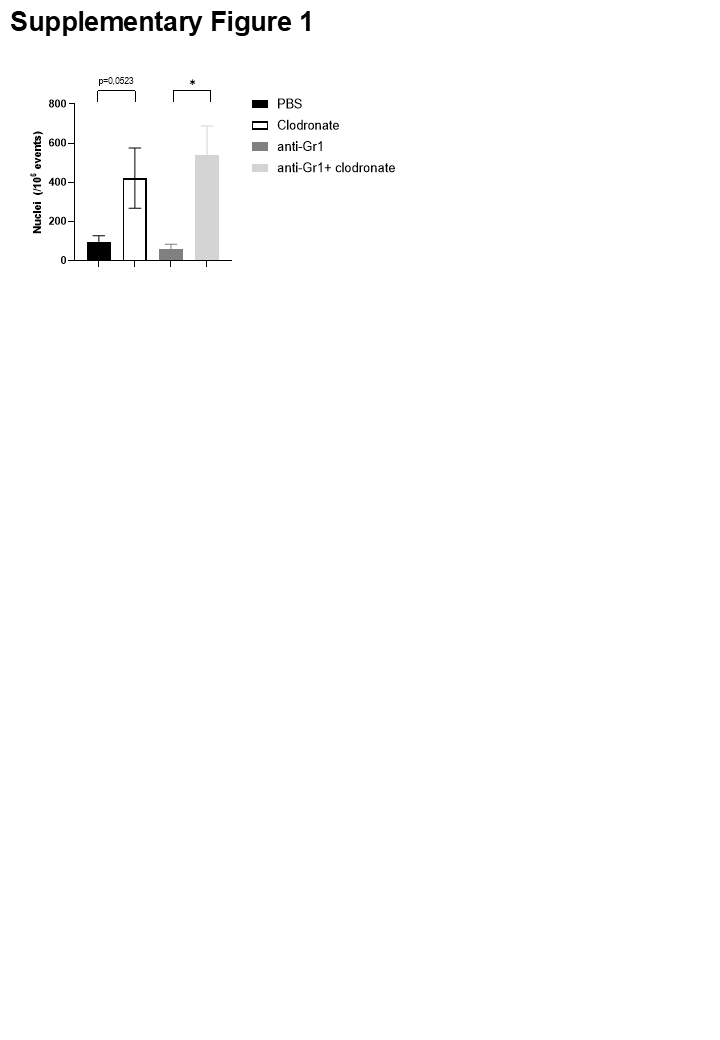

Supplement: Supplementary Figure 1 — The appearance of free nuclei particles was attributed only to depletion of macrophages and not of neutrophils and monocytes. C57BL/6 mice were treated intraperitoneally with 250 µl of clodronate liposomes in order to deplete macrophages and monocytes 24 hours before measurement of free nuclei particles by flow cytometry (n=3). 100 μg per mouse of anti-Gr-1 antibody (anti-mouse Ly6G/Ly6C antibody, clone RB6-8C5, Bio X Cell, Lebanon, USA) were applied i.v. for depletion of neutrophil granulocytes and monocytes 48 hours before measurement of free nuclei by flow cytometry (n=3). Results are presented as mean ± SEM. [file Image1.tif]

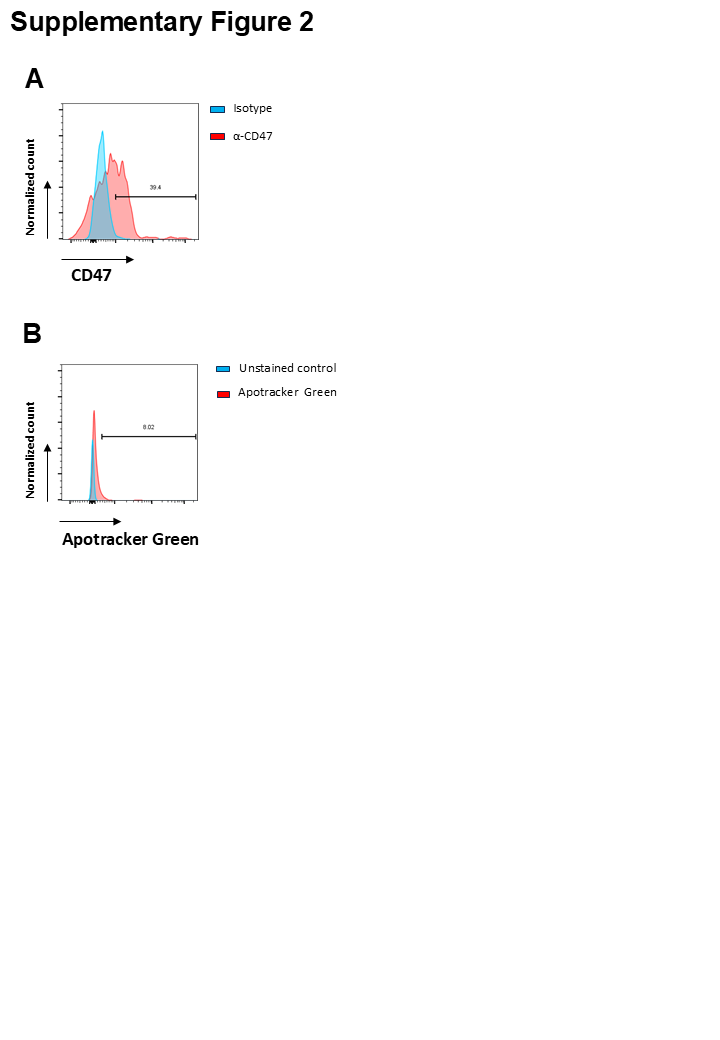

Supplement: Supplementary Figure 2 — Free nuclei were unlikely to represent apoptotic debris, as indicated by mild CD47 expression and low Apotracker Green positivity. (A) The whole blood of C57BL/6 mice pretreated at day -1 with 250 µl of clodronate liposomes was stained using an antibody against surface protein CD47, which is involved in inhibition of phagocytosis. The representative histogram shows positivity for CD47 of the population of free small sized, DAPI positive nuclei particles. Isotype antibody was used as control. (B) The whole blood of C57BL/6 mice pretreated at day -1 with 250 µl of clodronate liposomes was stained using Apotracker Green. The representative histogram shows positivity for Apotracker Green of the population of free small sized, DAPI positive nuclei particles. Unstained cells were used as negative control. [file Image2.tif]
